# Clinical Notes

**Published:** 1898-10

**Authors:** 


### CLINICAL NOTES.

Dr. R. R. Bell recommends for gastric tympanites large and frequent doses of animal charcoal, two parts, and bicarbonate of soda, one part, in capsule form.

Dr. S. S. Whitbeck advises aloes made up in bolus form with turpentine, given per rectum, in most instances getting response in from four to six hours. Tincture of aconite in full-sized doses for fermentation.

Dr. W. L. Williams strongly urges the relief of cæcal distention with the trochar and canula, and thus control gastric tympany and its disastrous sequences.

Dr. L. A. Merillat recommends strongly the use of eserine in those cases of acute indigestion where flatus is present and slight elimination of the same is noted.

Dr. M. H. Reynolds urges the use of strychnine and atropine in moderate doses in connection with eserine.

Dr. W. C. Langdon, State Veterinarian of North Dakota, believes that he now has two horses cured of glanders by the continued use of injections of mallein.

